# Three-way Philadelphia translocation t(9;10;22)(q34;p11.2;q11.2) as a secondary abnormality in an imatinib mesylate-resistant chronic myeloid leukemia patient

**DOI:** 10.3892/ol.2013.1228

**Published:** 2013-03-05

**Authors:** WALID AL-ACHKAR, ABDULSAMAD WAFA, ADNAN IKHTIAR, THOMAS LIEHR

**Affiliations:** 1 Department of Molecular Biology and Biotechnology, Human Genetics Division, Flow-cytometry Lab, Atomic Energy Commission, Damascus, Syria;; 2 Department of Molecular Biology and Biotechnology, Mammalian Biology Division, Flow-cytometry Lab, Atomic Energy Commission, Damascus, Syria;; 3 Jena University Hospital, Institute of Human Genetics, Jena, Germany

**Keywords:** chronic myeloid leukemia, three-way Philadelphia translocation, fluorescence *in situ* hybridization, multicolor banding, imatinib mesylate

## Abstract

Chronic myelogenous leukemia (CML) is characterized by the Philadelphia (Ph) chromosome created by the reciprocal translocation t(9:22)(q34;q11), resulting in the chimeric gene breakpoint cluster region (BCR)-Abelson (ABL). Variant Ph chromosome translocations involving chromosomes other than 9 and 22 occur in 5–10% of CML cases. In the present study, a novel case of a Ph chromosome-positive CML in the chronic phase (CP) is reported, with a three-way Ph translocation involving three chromosomal regions, 9q34, 10p11.2 and 22q11.2, in addition to the loss of the Y chromosome, where the latter was a secondary abnormality. Since the majority of CML cases are currently treated with imatinib, variant rearrangements generally have no specific prognostic significance, although the mechanisms involved in resistance to therapy have yet to be investigated. The underlying mechanisms and prognostic implications of these cytogenetic abnormalities are discussed in the present study.

## Introduction

Chronic myeloid leukemia (CML) is a clonal disorder characterized by the Philadelphia chromosome (Ph). The Ph chromosome results from a reciprocal translocation between the long arms of chromosomes 9 and 22 and is demonstrable in all hematopoietic precursors (1). This translocation results in the transfer of the Abelson (ABL) proto-oncogene on chromosome 9 to an area of chromosome 22 named the breakpoint cluster region (BCR). This results in a fused BCR/ABL gene and the production of an abnormal tyrosine kinase protein that causes the disordered myelopoiesis observed in CML. Imatinib mesylate (IM; Glivec), formerly STI57 (1), was the first available BCR/ABL-targeted therapy and produced complete cytogenetic responses in 70–85% of patients with CML in the early chronic phase (CP) (2). However, despite the remarkable efficacy of this agent, resistance or intolerance to IM has also been observed. Moreover, IM does not completely eradicate residual leukemic stem cells and progenitors (3).

In 5–10% of CML cases, variant rearrangements involving 9q34, 22q11.2 and one or more additional genomic regions generate the previously mentioned chimeric gene (4). Occasionally, the chromosome changes are submicroscopic so the translocation may be masked and revealed only by fluorescence *in situ* hybridization (FISH) or molecular analysis (5). The present study reports a rare case of Ph chromosome-positive CML in CP with variant translocation involving chromosome 10 at sub-band region p11.2, as well as 9q34 and 22q11.2. The rearrangement was characterized by FISH, multicolor banding (MCB) and reverse transcription polymerase chain reaction (RT-PCR).

## Materials and methods

### Case report

A 42-year-old male patient was diagnosed with CML in CP in February 2006. Hepatosplenomegaly, fever and loss of weight were the indicative symptoms. The patient then received IM at 400 mg/day intermittently for two years overall. However, in January 2011 the patient exhibited less marked symptoms. The patient’s hematological parameters were white blood cells (WBC) at 306×10^9^/l, consisting of 78% neutrophils, 18% lymphocytes, 1% monocytes and 1% basophiles. The platelet count was 500×10^9^/l and the hemoglobin level was 9.3 g/dl. The serum lactate dehydrogenase (LDH) level was 658 U/l (normal level up to 240 U/l).

The study was approved by the Biosafety and Bioethics Commitee of Syrian Atomic Energy Commssion. Written informed consent was obtained from the patient.

### Chromosome cytogenetics

Chromosome analysis was performed using the GTG-banding technique according to standard procedures (6). A total of 20 metaphase cells derived from unstimulated bone marrow culture were analyzed. Karyotypes were described according to the International System for Human Cytogenetic Nomenclature (7).

### Molecular cytogenetics

FISH using an LSI BCR/ABL dual-color dual-fusion translocation probe (Abbott Molecular/Vysis, Des Plaines, IL, USA) was used according to the manufacturer’s instructions (6). MCB probe sets based on microdissection-derived region-specific libraries for chromosomes 9, 10 and 22 were used as described previously (8). A minimum of 20 metaphase spreads were analyzed, using a fluorescence microscope (AxioImager.Z1 mot; Zeiss, Jena, Germany) equipped with appropriate filter sets to discriminate between a maximum of five fluorochromes and the counter-stain 4’,6-diamino-2-phenylindole (DAPI). Image capturing and processing were performed using an Isis imaging system (MetaSystems, Altlussheim, Germany).

### RT-PCR for BCR/ABL fusion transcripts

RT-PCR was performed as described previously (9).

### Immunophenotyping

Immunophenotyping of leukemic blasts was performed as described previously (10).

## Results

**Figure 1:**
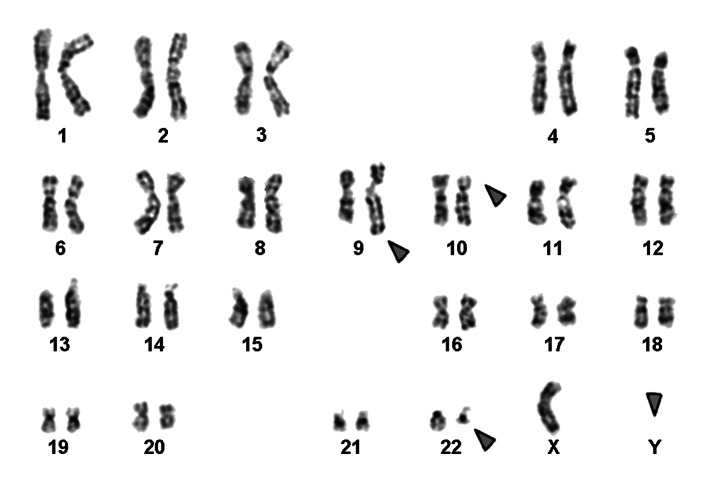
GTG-banding revealed a complex karyotype involving three chromosomes as well as the loss of the Y chromosome. All derivative chromosomes are highlighted by arrow heads.

**Figure 2:**
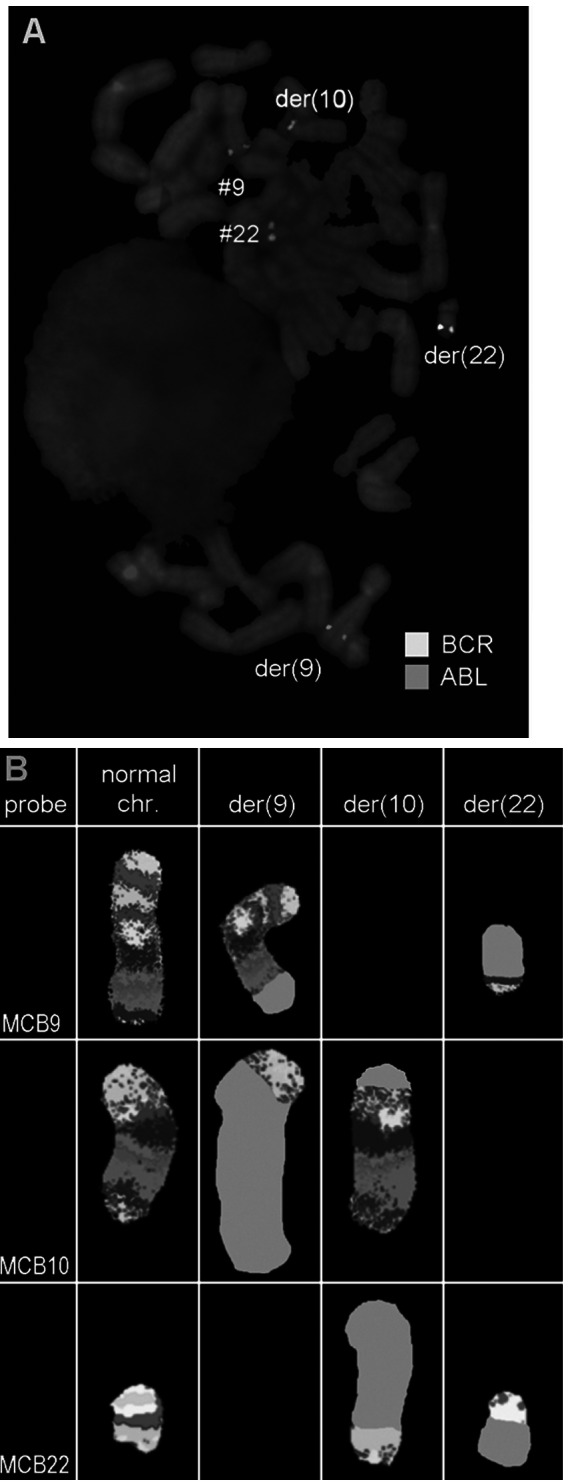
Karyotype and chromosomal aberrations were confirmed using molecular cytogenetic approaches. (A) Metaphase FISH using probes for BCR (white) and ABL (gray) confirmed an involvement of chromosome 10 in the rearrangement, the presence of the BCR/ABL translocation and the Ph chromosome. (B) MCB was applied to determine which chromosomes were involved in the complex rearrangement. Each image shows the results of MCB analysis using probe sets for chromosomes 9, 10 and 22. The normal chromosomes are shown in the first column and the derivatives of the three chromosomes in the subsequent columns. The MCB-probe unstained regions on the derivative chromosomes are shown in gray. #, chromosome; der, derivative chromosome; Ph, Philadelphia chromosome; FISH, fluorescence *in situ* hybridization; BCR, breakpoint cluster region; ABL, Abelson; MCB, multicolor banding.

Karyotyping was performed prior to and following the chemotherapy treatment. Initially, GTG-banding revealed 46,XY,t(9;22)[20]. The result following chemotherapy was 46,XY,t(9;10;22)[12]/45,X,-Y,t(9;10;22)[8] (Fig. 1), which was further specified by molecular cytogenetic studies (Fig. 2). Dual-color FISH using a probe specific for BCR and ABL revealed a typical Ph chromosome with the BCR/ABL fusion gene. However, sections of chromosome 22 were present on the derivative chromosome 10 [der(10)] (Fig. 2A). RT-PCR demonstrated a p210, b2a2 fusion transcript, most often observed in CML (data not shown). After the application of MCB, a complex translocation among chromosomes 9, 10 and 22 was revealed (Fig. 2B): 46,XY,t(9;10;22)(q34;p11.2;q11) [12]/45,X,-Y,t(9;10;22)(q34;p11.2;q11)[8].

Immunophenotyping analysis of the peripheral blood showed abnormal antigen expression profiles for CD16 (66.8%), CD64 (66.8%), CD32 (66.8%), CD33 (35%) and CD13 (84%). A high percentage of these cells were CD123-positive (20%). These cells were CD15 (85%), CD38 (62.7), CD11c (53%), CD10 (31%) and CD34 (2.9%). This immunophenotype was consistent with CML according to the FAB classification.

## Discussion

As reported previously, variant Ph translocations involving chromosome 10 are rare and only a few cases involving the short arm of chromosome 10 have been described. Furthermore, only one of these previously reported CML cases involved the breakpoint region 10p11 (11). To the best of our knowledge, the present study is the first to observe a case of Ph chromosome-positive CML in CP with t(9;10;22)(q34;p11.2;q11). Notably, there was another Ph-positive clone with a loss of chromosome Y secondary abnormality which most likely resulted in the resistance to IM chemotherapy (12).

Two possible mechanisms have been suggested for the formation of variant translocations (13). The first is a single event rearrangement via the simultaneous breakage of several chromosomes followed by mismatched joining. The other is a multi-step mechanism in which a classical Ph translocation is followed by further translocation events involving chromosomes 9 and 22, as well as other chromosomes. A WCP-FISH study demonstrated the insertion of chromosomal material from chromosome 22 into a variant chromosome. In this pattern, the BCR/ABL fusion gene was located on derivative chromosome 22. This suggests that three breaks have occurred on chromosomes 9, 22 and the variant chromosome and that 9q34∼qter was rejoined 22q11 at the same time as 22q11∼qter was inserted into the variant chromosome. This pattern is indicative of a one-step formation mechanism.

The progression of CML from the CP to blast crisis is frequently associated with non-random secondary chromosomal aberrations such as +8, i(17q), +19 and an extra Ph chromosome. Other less frequent abnormalities include -7, -17, -Y, +17 and +21 (14).

Resistance to chemotherapy may occur as a result of increased expression of the BCR-ABL kinase from genomic amplification, clonal chromosomal evolution or mutations in the ABL kinase of the BCR-ABL gene affecting drug interactions or kinase activity (15).

ABI-1 is located at 10p11.2 in humans and is a homolog to the mouse Abelson interactor 1 gene (Abi-1) It is one of the major regulators of actin cytoskeleton reorganization but its role in cancer progression and metastasis remains ill-defined (16). While Abi-1 (which has high homology to ABI-2) and ABI-2 are regulators of ABL function in transformation or signal transduction, the functional correlation between Abi-1, ABI-2 and BCR-ABL remains unknown (17).

Variant Ph translocation involving chromosome 10 may represent a more aggressive marker in CML and its presence may suggest an increased dose of IM as commonly administered in the accelerated phase (18).

In conclusion, the present study reported a Ph chromosome-positive CML case in the CP with a rare variant translocation involving chromosome 10p11.2 as well as 9q34 and 22q11.2 and the loss of a Y chromosome as a secondary abnormality which most likely resulted in the resistance to IM chemotherapy.
